# Identification of consensus homozygous regions and their associations with growth and feed efficiency traits in American mink

**DOI:** 10.1186/s12863-024-01252-8

**Published:** 2024-07-10

**Authors:** Pourya Davoudi, Duy Ngoc Do, Bruce Rathgeber, Stefanie Colombo, Mehdi Sargolzaei, Graham Plastow, Zhiquan Wang, Younes Miar

**Affiliations:** 1 Department of Animal Science and Aquaculture, Dalhousie University, Truro, NS Canada; 2 Department of Pathobiology, University of Guelph, Guelph, ON Canada; 3 Select Sires Inc, Plain City, OH USA; 4 Department of Agricultural, Food and Nutritional Science, Livestock Gentec, University of Alberta, Edmonton, AB Canada

**Keywords:** Runs of homozygosity, Feed efficiency, Growth traits, Association analysis, American mink

## Abstract

The recent chromosome-based genome assembly and the newly developed 70K single nucleotide polymorphism (SNP) array for American mink (*Neogale vison*) facilitate the identification of genetic variants underlying complex traits in this species. The objective of this study was to evaluate the association between consensus runs of homozygosity (ROH) with growth and feed efficiency traits in American mink. A subsample of two mink populations (*n* = 2,986) were genotyped using the Affymetrix Mink 70K SNP array. The identified ROH segments were included simultaneously, concatenated into consensus regions, and the ROH-based association studies were carried out with linear mixed models considering a genomic relationship matrix for 11 growth and feed efficiency traits implemented in ASReml-R version 4. In total, 298,313 ROH were identified across all individuals, with an average length and coverage of 4.16 Mb and 414.8 Mb, respectively. After merging ROH segments, 196 consensus ROH regions were detected and used for genome-wide ROH-based association analysis. Thirteen consensus ROH regions were significantly (*P* < 0.01) associated with growth and feed efficiency traits. Several candidate genes within the significant regions are known for their involvement in growth and body size development, including *MEF2A*, *ADAMTS17*, *POU3F2*, and *TYRO3*. In addition, we found ten consensus ROH regions, defined as ROH islands, with frequencies over 80% of the population. These islands harbored 12 annotated genes, some of which were related to immune system processes such as *DTX3L*, *PARP9*, *PARP14*, *CD86*, and *HCLS1*. This is the first study to explore the associations between homozygous regions with growth and feed efficiency traits in American mink. Our findings shed the light on the effects of homozygosity in the mink genome on growth and feed efficiency traits, that can be utilized in developing a sustainable breeding program for mink.

**Supplementary Information:**

The online version contains supplementary material available at 10.1186/s12863-024-01252-8.

## Introduction

American mink breeding is entering the genomic era through the availability of a high-quality chromosome-based genome assembly [1] and a genome-wide single-nucleotide polymorphisms (SNPs) array. Such technologies have paved the way for the precise identification of homozygous segments in livestock species [2]. Runs of homozygosity (ROH) are homozygous regions, which are composed of two identical haplotypes inherited from a common ancestor [3]. Characteristics of ROH in a population can be used as an indicator for estimation of inbreeding level in different species, such as cattle [4, 5], pigs [6, 7], chicken [8, 9], sheep [10, 11], goat [12, 13], and buffalo [14, 15].

Groups of several ROH within a specific region of the genome in a population are known as ROH islands [16]. It was reported that the analysis of ROH islands might reveal genomic regions under selection pressure, which in turn helps to identify candidate genes associated with traits of economic interest [17, 18]. Furthermore, several studies have suggested the feasibility of performing association analyses using ROH to detect homozygous genomic regions associated with complex traits in livestock [19]. Sanglard et al. [20] identified several regions of ROH significantly associated with antibody response to porcine reproductive and respiratory syndrome virus vaccination in pigs. In cattle, substantial numbers of ROH regions are reported to be associated with milk yield [21, 22], fertility [23, 24], and production traits [25], suggesting a complementary role of ROH in elucidating the genetic mechanisms underlying economically important traits.

Feed cost is the largest expense for mink production systems, and thereby, improving feed efficiency holds significant potential for increasing the profitability of mink farming through strategic breeding programs [26]. Several studies have reported moderate to high heritability for growth [27, 28] and feed efficiency traits [26, 29, 30] in American mink, which highlighted a substantial genetic basis and presented opportunities for improvement by genetic and genomic breeding programs.

To the best of our knowledge, there is no study that examined homozygous segments in the American mink genome and their potential association with growth and feed efficiency traits. Therefore, the main objectives of this study were to (1) reveal the distribution and pattern of ROH within the genome of American mink; (2) identify highly frequent consensus ROH (ROH islands) and investigate the candidate genes within these regions; and (3) assess their associations with growth and feed efficiency traits.

## Materials and methods

### Animals and traits

Mink were humanely euthanized using carbon monoxide sourced from a compressed gas cylinder, adhering to the protocols outlined in the Canada Mink Breeders Association’s Code of Practice for the Care and Handling of Farmed Mink (ISBN 978-1-988793-24-5) (https://www.nfacc.ca/codes-of-practice/farmed-mink). The procedure involved maintaining a minimum of 4% carbon monoxide concentration within the chamber to ensure a swift and irreversible onset of unconsciousness, leading to a quick and relatively painless death for the mink. Confirmation of the mink’s death was conducted through a thorough check for the cessation of vital signs, which included no movement, the absence of heartbeat and respiration, pupils that were fixed and dilated, and a lack of reflexive responses.

A detailed description of the phenotypic data used in this study can be found in Davoudi et al. [26]. Briefly, a total of 2,288 American mink with growth and feed efficiency records were available. These traits were collected according to Davoudi et al. [26]: final body weight (FBW), final body length (FBL), harvest weight (HW), harvest length (HL), daily feed intake (DFI), average daily gain (ADG), feed conversion ratio (FCR), Kleiber ratio (KR), residual feed intake (RFI), residual gain (RG), and residual intake and gain (RIG). Descriptive statistics for growth and feed efficiency in American mink are shown in Additional file 1: Table S1.

### SNP genotyping and quality control

All mink were genotyped using the Affymetrix Mink 70K SNP array (Neogen, Lincoln, Nebraska, United States). Genotypes were pruned by PLINK 1.9 software based on the proportion of missing genotypes (> 0.95), individual call rate (> 0.90), and Hardy-Weinberg equilibrium (*P* > 10e-6). In addition, SNPs located on sex chromosomes were removed, resulting in a final data set of 49,268 SNPs for further analyses.

### Assessment of runs of homozygosity

We used PLINK 1.9 software [31] to identify homozygous segments across autosomes of each individual’s genome. The ROH were discovered based on the sliding window approach with the following parameters: (1) sliding window of 50 SNPs across the genome; (2) a minimum ROH length of 1,000 kb; (3) the minimum SNP density was set to 50 kb/SNP; (4) maximum gap between consecutive homozygous SNPs was 1,000 kb; (5) only one heterozygous and one missing genotype were allowed; and (6) a minimum of 57 consecutive SNPs were included in an ROH, which was determined according to the formula proposed by Lencz et al. [32], to control the false positive rate of the identified ROH:

$$The\, minimal\, number\, of\, SNPs\, in\, an\, ROH=\frac{{log}_{e}\frac{\alpha }{{n}_{a}{n}_{s}}}{{log}_{e}(1-het)}$$,

where $\alpha$ is the percentage of false positive ROH (set to 0.05), ${n}_{s}$ is the number of genotyped SNPs per individual, ${n}_{a}$ is the number of individuals, and $het$ is the proportion of heterozygosity across all SNPs.

### Consensus regions and ROH islands

The ‘*homozyg-group*’ function of the PLINK 1.9 software [31] was applied to merge the individual ROH into different ROH groups in a pool containing the overlapping regions between all the individual ROH in the group i.e. the consensus homozygous region [25, 33]. We retained the consensus ROH with a minimum of five SNPs and a frequency of more than 5% for association analyses. In addition, to investigate the genomic regions with a high frequency of ROH in the population (ROH islands), a threshold of higher than 80% was defined for consensus ROH [34]. The overlapped genes within ROH islands were annotated from the American mink reference genome annotation file [1] through the ‘*intersect*’ function in Bedtools version 2.30.0 [35].

### Association analyses between consensus ROH and phenotypes

According to the model described by Sanglard et al. [20], we evaluated the association between consensus ROH with growth and feed efficiency traits using the linear model as follows:

$$y=\mu +Xb+Zu+e,$$

where $\varvec{y}$ is the vector of phenotypic observation, $\mu$ is the grand mean, $\varvec{b}$ is the vector of fixed effects, $\varvec{X}$ and $\varvec{Z}$ are the incidence matrices that relate the fixed and random effects with the dependent variable, respectively; $\varvec{u}$ is the vector of random animal genetic effects and **e** is the vector of random residual effects. The random effects $\varvec{u}$ and $\varvec{e}$ were distributed as: $\varvec{u} \sim N(0, \varvec{G}{\sigma }_{u}^{2})$ and $\varvec{e} \sim N(0,\varvec{I}{\sigma }_{e}^{2})$, where ${\sigma }_{u}^{2}$ and ${\sigma }_{e}^{2}$ are the additive genetic and residual variances, respectively, **G** is the genomic relationship matrix, which was constructed by ASRgenomics package [36] using the VanRaden Eq. [37], and$\varvec{I}$is an identity matrix. The consensus ROH (*n*=196) were simultaneously fitted in the model as categorical fixed effects, coding as “yes” if the individual contained the ROH segment, or “no” otherwise. The other fixed effects, as described by [26], were farm (two farms), sex (male and female), color type (dark, demi, mahogany, pastel, and stardust), row-year (year: 2018 and 2019; row: 1, 4, 5, 7, 8, and 11). We included color type in our analyses because it significantly impacts growth parameters in American mink, likely due to the pleiotropic effects of genes controlling both feed efficiency and color type [26]. The age of animals (in days; with a minimum and maximum of 184 and 229, respectively) was included as a covariate in the model. The fixed effects and covariate were statistically tested (*P* < 0.01) using univariate models in ASReml 4.0 [38]. The associations between each consensus ROH and studied traits were tested through linear mixed model analysis in ASReml 4.0 [38] with a statistical significance level (*P* < 0.01).

## Results

### Assessment of runs of homozygosity

**Fig. 1:**
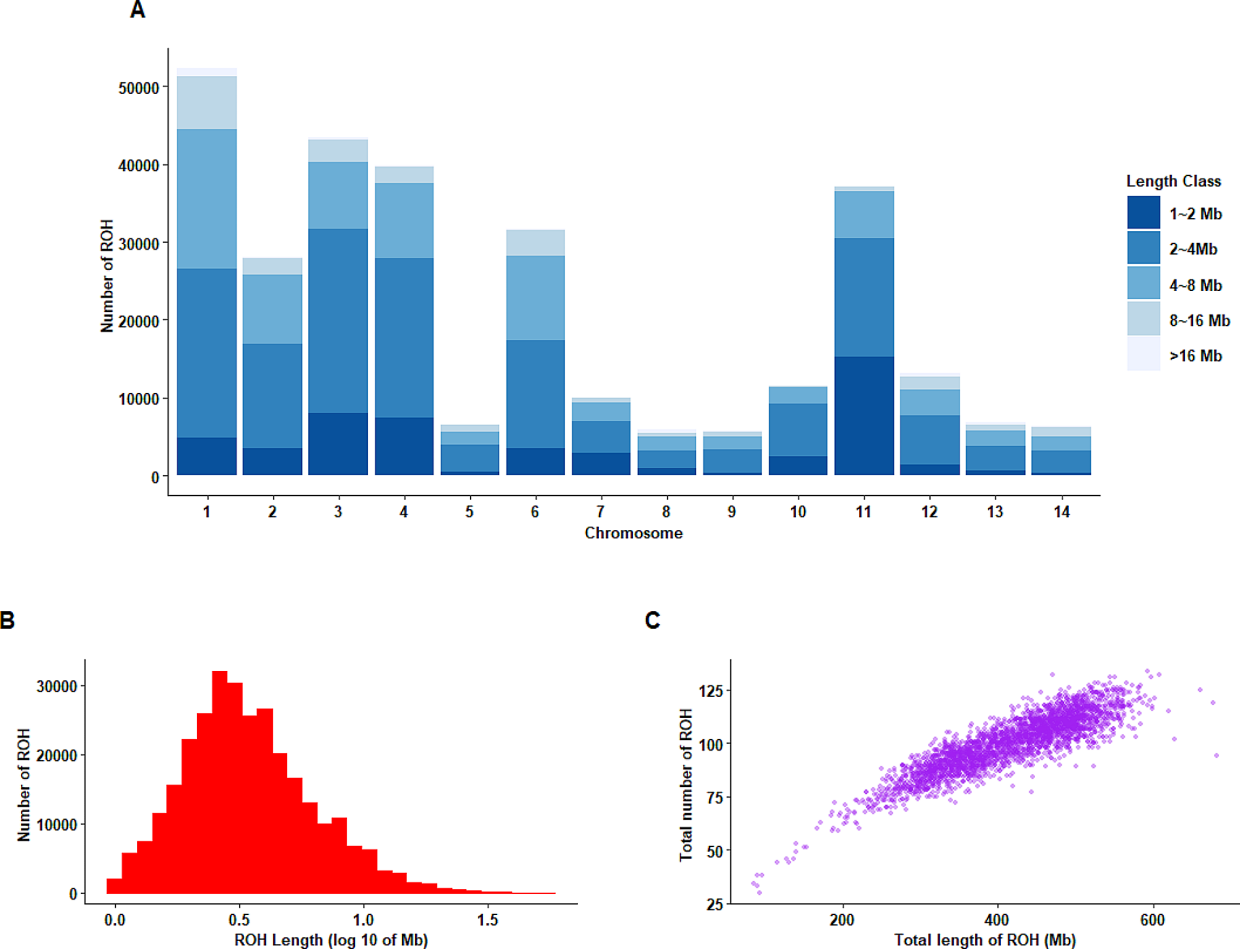
Characteristics of runs of homozygosity in American mink: **(A)** Frequency distribution of the average number of ROH in different length classes (Mb) in each chromosome; **(B)** Length distribution of ROH; **(C)** Relationship between ROH number per animal and total length of the genome covered by them. Each point represents one individual

A total of 298,313 runs of homozygosity (ROH) were identified in the entire mink population studied. Detailed information on detected ROH in all individuals is provided in Additional file 1: Table S2. The results showed that the average number of ROH segments per individual was 99.90, spanning from 30 to 134, respectively, and the length of ROH segments ranged from 1.02 to 55.44 Mb, with an average of 4.16 Mb (Table 1). We classified ROH segments into five different length categories, including 1–2 Mb, 2–4 Mb, 4–8 Mb, 8–16 Mb, and > 16 Mb (Fig. 1A). The majority of detected ROH were classified as 2–4 Mb, representing 46.99% of all ROH (*n* = 140,178), followed by the length of 4–8 Mb and 1–2 Mb with 26.3% (*n* = 78,451) and 17.42% (*n* = 51,967), respectively. The percentage of ROH segments higher than 16 Mb was only 1.08% of all detected ROH (*n* = 3,236). The distribution of ROH lengths across the genome is represented in Fig. 1B. The largest ROH was located on chromosome 1 (55.44 Mb with 1563 SNPs), and the shortest ROH was identified on chromosome 3 (1.02 Mb with 79 SNPs). Further, the number of ROH segments varied across chromosomes, ranging from the lowest in chromosome 9 (*n* = 5,728) to the highest in chromosome 1 (*n* = 52,311). As shown in Fig. 1C, the total length of the genome covered by ROH among individuals ranged from 84.78 Mb to 683.16 Mb, with an average of 414.81 Mb.

**Table 1:** Descriptive statistics of runs of homozygosity (ROH) number and length by ROH length class

Class	Number	Percentage (%)	Average size (Mb)	Standard Deviation (Mb)
1–2 Mb	51,967	17.42	1.59	0.28
2-4 Mb	140,178	46.99	2.89	0.56
4–8 Mb	78,451	26.3	5.44	1.14
8–16 Mb	24,481	8.21	10.49	2.02
> 16 Mb	3,236	1.08	21.03	4.87
Total	298,313	100	4.16	3.12

### Consensus regions and ROH islands

**Fig. 2:**
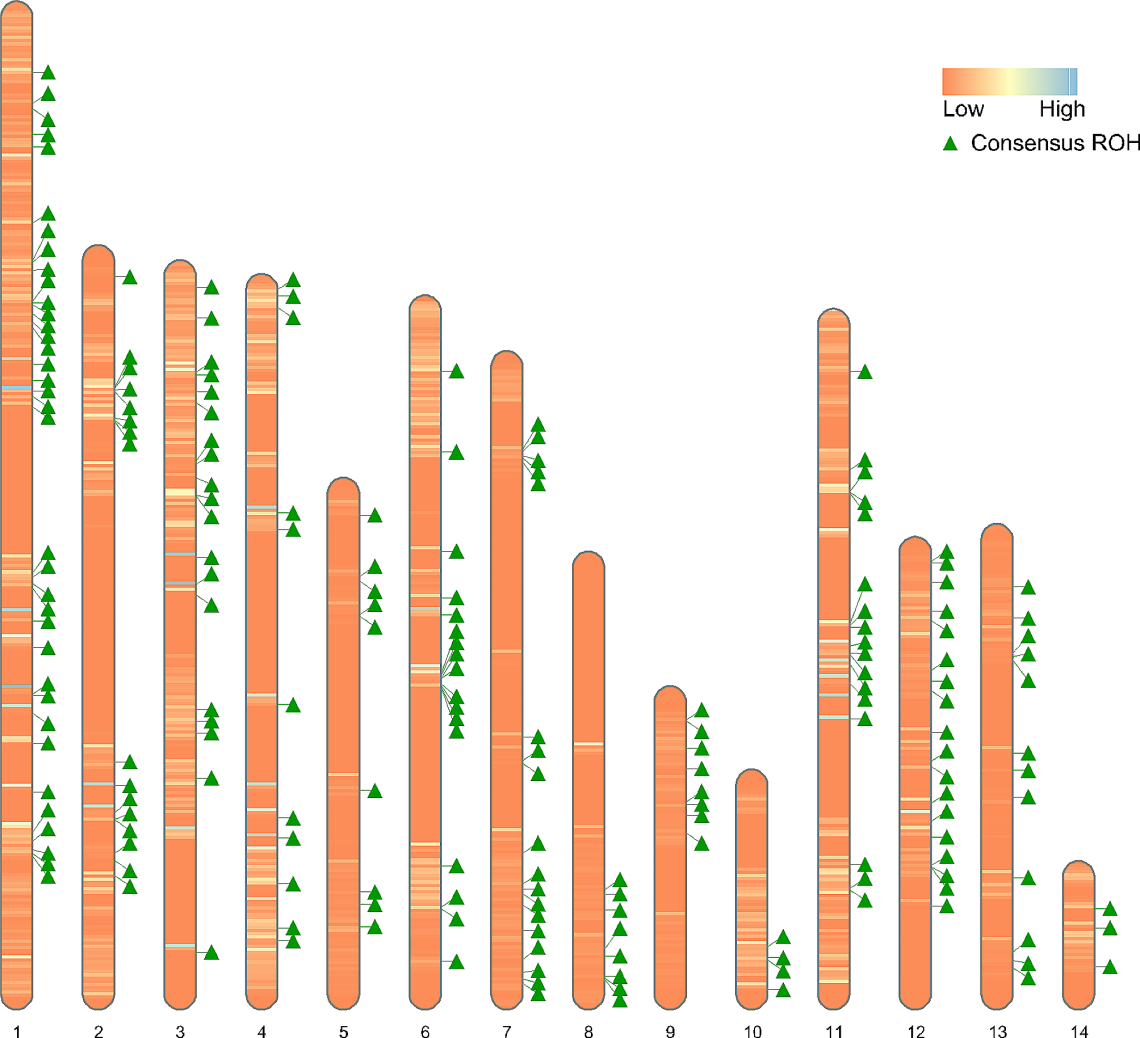
Chromosome ideograms showing the position of identified ROH and consensus ROH shared between individuals. The color scale within each chromosome represents the number of identified ROH, changing the gradient with more ROH detected in an area. The position of consensus ROH is marked with the green triangle next to the chromosome

To provide the shared homozygous regions for the association analyses, initially, 6,980 consensus groups were formed using ‘*–homozyg-group’* function in PLINK 1.9 software, of which a total of 196 consensus ROH fulfilled the criteria of presenting in more than 5% of individuals with a minimum of five SNPs (Additional file 1: Table S3). The chromosomal distribution map of identified ROH across mink autosomes and consensus ROH shared among individuals is shown in Fig. 2.

The ROH islands were determined as regions where the consensus ROH was presented in more than 80% of animals, with the aim of pinpointing the genes they encompass. The implementation of this approach resulted in the detection of ten ROH islands spanning 14 autosomes, most of which were located on chromosome six with seven ROH islands. These specific regions harbored 12 annotated genes, some with known effects on immune systems processes such as *DTX3L*,* PARP9*,* PARP14*,* CD86*, and *HCLS1* (Table 2). Notably, the three ROH islands on other chromosomes did not contain any known annotated genes.

**Table 2:** Characterization of consensus ROH shared by more than 80% of the mink population and the annotated genes in the corresponding regions

Chr		Start	End	Length (bp)	Frequency (%)^a^	No. SNPs	Annotated genes
6	122,500,609		122,510,002	9,394	86.24	5	*CD86*
6	122,846,667		122,881,153	34,487	84.63	6	*GOLGB1*
6	122,908,246		122,958,392	50,147	84.33	5	*FBXO40; GOLGB1; HCLS1*
6	100,386,881		100,611,871	224,991	82.92	14	*-*
6	121,883,426		122,139,161	255,736	82.02	6	*SLC49A4; PARP9; DTX3L; LOC122908877; PARP14; HSPBAP1; LOC122911033*
2	45,138,611		45,265,052	126,442	81.92	19	*-*
2	45,100,650		45,130,801	30,152	81.88	6	*-*
6	120,478,852		120,531,357	52,506	81.41	6	*KALRN*
6	120,546,042		120,564,621	18,580	81.41	5	*KALRN*
1	217,785,451		217,894,574	109,124	80.17	5	*LOC122897674*

^a^ Percentage of the population presented this ROH.

### Association analyses between consensus ROH and phenotypes

**Fig. 3:**
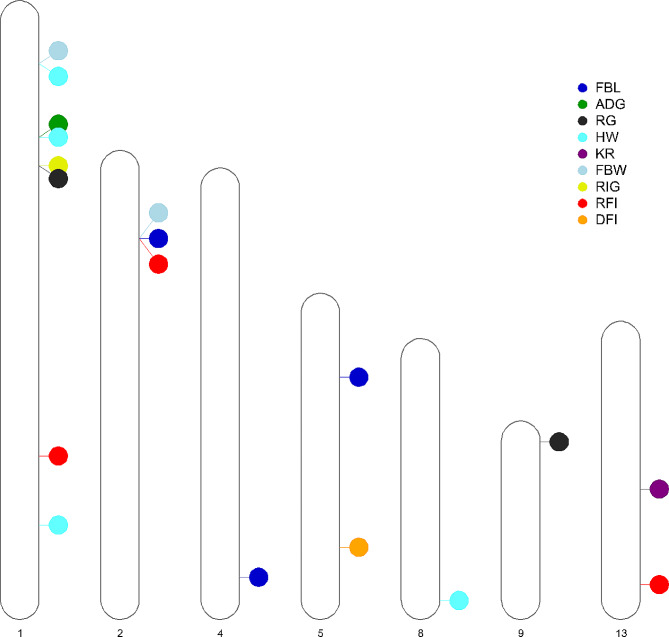
Physical position of significant consensus ROH across the mink autosomes. FBL: Final body length, ADG: Average daily gain, RG: Residual gain, HW: Harvest weight, KR: Kleiber ratio, FBW: Final body weight, RIG: Residual intake and gain, RFI: Residual feed intake, DFI: Daily feed intake

The association analysis revealed 13 consensus regions that were significantly (*P* < 0.01) associated with growth and feed efficiency traits, of which four ROH affected more than one trait. The physical position of significant consensus ROH across the mink autosomes is shown in Fig. 3. The frequency of the associated consensus regions ranged from 6.6 to 81.9% across all individuals. The average length of significant consensus ROH was 147.46 kb, ranging from 8.62 to 327.85 kb. Chromosome one exhibited the highest number of significant regions (*n* = 5), followed by two significant regions on chromosome 13, and one significant region on chromosomes 2, 4, 5, 8, and 9. Detailed information regarding the consensus ROH significantly associated (*P* < 0.01) with the studied traits, along with their annotated candidate genes can be found in Table 3.

**Table 3:** Regions of runs of homozygosity (ROH) significantly associated with growth and feed efficiency traits in American mink

Chr	Start	End	Length (bp)	Associated traits	*P*-value^a^	Frequency (%)^b^	No. SNPs	Candidate genes
1	233,051,034	233,378,886	327,853	RFI	0.0038	47.9	20	*PPP2R2B*
1	84,492,422	84,643,650	151,229	RG; RIG	0.0094; 0.0084	69.6	5	*MDGA1*
1	268,594,555	268,631,729	37,175	HW	0.0064	39.3	7	*THG1L*
4	20,9695,778	209,720,164	24,387	FBL	0.00027	66.6	5	*COPG2*
2	45,138,611	45,265,052	126,442	FBL; FBW; RFI	0.0014; 0.0016; 0.0065	81.9	19	*-*
5	42,989,622	43,198,887	209,266	FBL	0.0063	20.8	5	*-*
1	32,128,510	32,316,405	187,896	FBW; HW	0.0034; 0.0051	16.1	11	*POU3F2; FBXL4; LOC122913962*
5	130,209,831	130,237,085	27,255	DFI	0.0069	15.1	5	*KLHL1*
13	85,787,981	86,060,018	272,038	KR	0.0066	10.9	5	*MGA; OIP5; NUSAP1; RTF1; LTK; RPAP1; NDUFAF1; ITPKA; TYRO3; LOC122894302; LOC122894795; LOC122894805; LOC122894585*
9	10,609,898	10,786,369	176,472	RG	0.0023	10.9	6	*LHX2*
13	134,704,541	13,500,3083	298,543	RFI	0.0034	9.9	6	*LYSMD4; ADAMTS17; MEF2A*
8	134,262,073	134,331,827	69,755	HW	0.0062	6.6	5	*-*
1	69,893,506	69,902,125	8,620	HW; ADG	0.0047; 0.0065	55.1	7	*AKAP7*

^a^*P*-value < 0.01. ^b^ Percentage of the population presented this ROH.

## Discussion

In this study, the mean number of ROH per individual was 99.9, which was in agreement with Karimi et al. [1] who reported an average of 102 per animal using whole-genome sequencing data of 100 American mink. Yet, both studies reported higher numbers of ROH counts compared to the study of Karimi et al. [39], which identified 82 ROH segments per individual solely based on scaffolds. This discrepancy indicates that the recent chromosome-based reference genome in American mink has facilitated our capacity to detect homozygous segments. The distribution of detected ROH revealed that approximately more than 90% of ROH were shorter than 8 Mb, which was consistent with the results reported in other species, such as cattle [17, 40], pigs [7, 41], chicken [42, 43], sheep [44, 45], and buffalo [14, 46]. It is well-established that the large ROH (~ 10 Mb) represents recent inbreeding (up to five generations ago), whereas short ROH (~ 1 Mb) indicates more distant ancestral effects (up to 50 generations ago) [47, 48]. Considering the predominant of ROH with a length of 1 to 8 Mb, it is reasonable to hypothesize that the inbreeding events in American mink occurred approximately 5 to 50 generations ago. This timeline corresponds with the findings of Hu et al. (2023), who reported the rapid decline in the effective population size in American mink from 5 to 50 generations ago.

In recent years, the identification of ROH islands across the genome has gained popularity due to their capacity to reveal selection footprint in livestock species [49]. The Aleutian disease, the most significant health concern for global mink farming, is an immune complex disease that causes autoimmune disorders in mink [50]. Despite efforts to detect and eliminate infected animals using various immunological tests, these strategies have largely failed due to the high persistence nature of Aleutian disease in the breeding environment [51, 52]. Intriguingly, our study uncovered several genes within ROH islands known to affect immune system processes, including *DTX3L*,* PARP9*,* PARP14*,* CD86*, and *HCLS1*. This implies that natural selection plausibly acts on immune-related genes in American mink.

The *DTX3L* gene, also known as *BBAP* (B-lymphoma and BAL-associated protein), plays regulatory functions on DNA damage signaling, tumor cell growth, and IFN signaling and antiviral response [53–55]. Interestingly, Hong et al. [56] reported that inhibiting the *DTX3L* gene restrained the cell invasion and secretion of inflammatory factors, suggesting its potential as a therapeutic target for rheumatoid arthritis, a complex autoimmune disease characterized by chronic synovitis of the joints in humans. The *PARP9* and *PARP14* genes, located within the ROH island on chromosome 6 (121,883,426:122,139,161 bp), belong to the PARP superfamily that regulate diverse biological processes such as DNA damage repair, cellular stress response, and antiviral innate immunity [57]. Research has demonstrated that *PARP9* gene, highly expressed in glioma, is correlated with checkpoint molecules involved in inflammatory and immune responses [58]. Moreover, study has shown that knockdown of *PARP9* gene in human or mouse dendritic cells and macrophages resulted in substantial reduction of type I IFN production (*IFN-α* and *IFN-β*), highlighting its critical role in the antiviral immunity system [59]. Similarly, *PARP14* knockout has shown therapeutic effects on tumors and allergic inflammation through mediating T-cell differentiation and action of cytokines [60, 61]. Other genes of interest were *CD86* and *HCLS1* located within two different ROH islands on chromosome six (122,500,609:122,510,002 bp and 122,908,246:122,958,392 bp, respectively). Several lines of evidence indicated that *CD86*, which is one of the essential co-stimulatory molecules expressed on antigen presenting cells, plays a regulatory role in the immune response by mediating the activation of T-cells, B-lymphocytes, and macrophages [62, 63]. It was indicated that the *HCLS1* gene, which is expressed only in cells with lymphohematopoietic origin, plays a functional role in the regulation of T-cell immune synapses [64].

It is well-documented that American mink is one of the most highly susceptible non-human species to severe acute respiratory syndrome coronavirus-2 (SARS-CoV-2) infection, leading to massive culls of many millions of mink across the world [65–67]. Intriguingly, most of the aforementioned genes, one way or another, have been reported to be associated with SARS-CoV-2, the virus that causes coronavirus disease-2019 (COVID-19). It was indicated that in SARS-CoV-2 infection, the activation of macrodomain-sensitive ADP-ribosylation signal is mediated by *PARP9*/*DTX3L* complex, suggesting their critical role in interferon-mediated antiviral defence [68]. Similarly, it was reported that the *PARP14* gene is essential for the optimal IFN expression, supporting the suggestion that *PARP14* is involved in antiviral immune response in CoV-infected cells [69]. Several studies have shown that the expression of *CD86* on monocytes and dendritic cells was substantially decreased in patients with severe COVID-19 [70–73]. These findings merit further exploration of the functional role of the ROH islands-harbored genes revealed in the current study on the Aleutian mink disease virus and COVID-19 infection in American mink.

In the present study, gene discovery performed on the 13 consensus regions that were significantly (*P* < 0.01) associated with growth and feed efficiency traits, highlighted several candidate genes (i.e. *MEF2A*,* ADAMTS17*,* POU3F2*, and *TYRO3*) with potential impacts on growth rate and feed efficiency as reported in previous studies. The *MEF2A* and *ADAMTS17* were located within the consensus ROH on chromosome 13 (134,704,541:135,003,083 bp), which was significantly (*P* < 0.01) associated with RFI. The *MEF2A* gene, which plays an important role in vertebrate skeletal muscle development and differentiation by activation of numerous muscle-specific and growth factor-induced genes [74], is known to be the candidate gene for muscle development and body growth in livestock species [75–77]. Remarkably, research conducted by Foroutan et al. [78] revealed that *MEF2A* showed higher expression levels across all tested tissues (liver, muscle, and testis) in the offspring of low-RFI Angus bulls, as opposed to their high-RFI counterparts. The *ADAMTS17* gene, which is a member of ADAMTS proteins with numerous biological functions [79], has been previously reported as one of the height-associated variants in several species, such as horse [80], cattle [81], dog [82, 83], and human [84–86]. Interestingly, the *ADAMTS17* gene was reported as a selective signal associated with animal height in the Shetland pony [87], and Brazilian locally adapted taurine cattle [88], highlighting the potential impacts of *ADAMTS17* gene on body size.

The *POU3F2* gene located within a ROH on chromosome 11 (32,128,510: 32,316,405 bp), is associated with HW and FBW traits. The *POU3F2* gene, which is widely expressed in the central nervous system, has been well-described to play a key role in diverse neuronal functions and hormonal regulation [89, 90]. Notably, Schönauer et al. [91] reported a negative correlation of *POU3F2* gene expression with body mass index in humans, suggesting the critical role of *POU3F2* in hyperphagic obesity in humans. The *TYRO3* gene was found within the consensus ROH on chromosome 13 (85,787,981: 86,060,018 bp), significantly associated with the KR trait. The *TYRO3* gene, which is expressed in neurons of the central nervous system, plays regulatory roles in cell proliferation and differentiation, associating with adipocyte size in moderately obese individuals [92]. A GWAS analysis by Sun et al. [93] reported that *TYRO3* gene was associated with intramuscular fat content in the breast muscle of chicken. Interestingly, it was revealed that *TYRO3* was significantly differentially expressed in muscle between low and high RFI pigs, indicating that *TYRO3* might affect the body fat, and consequently increase feed efficiency in pigs [94].

## Conclusion

We characterized the distribution of ROH and ROH islands, and the association between the consensus ROH with growth and feed efficiency traits in American mink. In total, we identified 13 consensus regions significantly associated with the studied traits, harboring several candidate genes that are known to be associated with growth and body size development, such as *MEF2A*,* ADAMTS17*,* POU3F2*, and *TYRO3*. In addition, ten ROH islands were identified across the genome, harboring genes related to immune systems processes such as *DTX3L*,* PARP9*,* PARP14*,* CD86*, and *HCLS1*. Overall, the results revealed the impact of homozygosity in the mink genome on growth and efficiency traits. These findings have important implications for the evaluation and selection of American mink in genetic improvement programs, offering valuable insights for enhancing the breeding and sustainability of this species.

### Electronic supplementary material

Below is the link to the electronic supplementary material.

Supplementary Material 1

Supplementary Material 2

Supplementary Material 3

## Data Availability

The data that support the findings of this study are available on request from the corresponding author. The data are not publicly available due to privacy or ethical restrictions.

## References

[1] Karimi K, Do DN, Wang J, Easley J, Borzouie S, Sargolzaei M (2022). A chromosome-level genome assembly reveals genomic characteristics of the American mink (Neogale vison). Commun Biology.

[2] Peripolli E, Munari D, Silva M, Lima A, Irgang R, Baldi F (2017). Runs of homozygosity: current knowledge and applications in livestock. Anim Genet.

[3] Ceballos FC, Joshi PK, Clark DW, Ramsay M, Wilson JF (2018). Runs of homozygosity: windows into population history and trait architecture. Nat Rev Genet.

[4] Eriksson S, Strandberg E, Johansson AM (2023). Changes in genomic inbreeding and diversity over half a century in Swedish Red and Swedish holstein dairy cattle. J Anim Breed Genet.

[5] Falchi L, Cesarani A, Mastrangelo S, Senczuk G, Portolano B, Pilla F, Macciotta NPP. Analysis of runs of homozygosity of cattle living in different climate zones. J Anim Sci. 2023;101. 10.1093/jas/skad061 PMC10066727 36802370

[6] Wu X, Zhou R, Wang Y, Zhang W, Zheng X, Zhao G (2022). Genome-wide scan for runs of homozygosity in Asian wild boars and Anqing six‐end‐white pigs. Anim Genet.

[7] Jiang Y, Li X, Liu J, Zhang W, Zhou M, Wang J (2022). Genome-wide detection of genetic structure and runs of homozygosity analysis in Anhui indigenous and western commercial pig breeds using PorcineSNP80k data. BMC Genomics.

[8] Wang Q, Zhang J, Wang H, Wang Z, Li Q, Zhao G (2023). Estimates of genomic inbreeding and identification of candidate regions in Beijing-You chicken populations. Anim Genet.

[9] Yuan J, Li S, Sheng Z, Zhang M, Liu X, Yuan Z (2022). Genome-wide run of homozygosity analysis reveals candidate genomic regions associated with environmental adaptations of tibetan native chickens. BMC Genomics.

[10] Ghoreishifar SM, Moradi-Shahrbabak H, Parna N, Davoudi P, Khansefid M (2019). Linkage disequilibrium and within-breed genetic diversity in Iranian Zandi sheep. Archives Anim Breed.

[11] Abdoli R, Mirhoseini SZ, Ghavi Hossein-Zadeh N, Zamani P, Moradi MH, Ferdosi MH et al. Runs of homozygosity and cross-generational inbreeding of Iranian fat-tailed sheep. Heredity. 2023:1–10. 10.1038/s41437-023-00611-y PMC10238534 37016136

[12] Manunza A, Diaz JR, Sayre BL, Cozzi P, Bobbo T, Deniskova T (2023). Discovering novel clues of natural selection on four worldwide goat breeds. Sci Rep.

[13] Ziegler TE, Molina A, Ramón M, Sanchez M, Muñoz-Mejías E, Antonini A, Demyda‐Peyrás S (2023). Analysis of the genomic landscape of inbreeding in two divergent groups of Spanish Florida goats. J Anim Breed Genet.

[14] Ghoreishifar SM, Moradi-Shahrbabak H, Fallahi MH, Jalil Sarghale A, Moradi-Shahrbabak M, Abdollahi-Arpanahi R, Khansefid M (2020). Genomic measures of inbreeding coefficients and genome-wide scan for runs of homozygosity islands in Iranian river buffalo, Bubalus bubalis. BMC Genet.

[15] Liu S-h, Ma X-y, Hassan F-u, Gao T-y (2022). Deng T-x. Genome-wide analysis of runs of homozygosity in Italian Mediterranean buffalo. J Dairy Sci.

[16] Nothnagel M, Lu TT, Kayser M, Krawczak M (2010). Genomic and geographic distribution of SNP-defined runs of homozygosity in europeans. Hum Mol Genet.

[17] Rocha RFB, Garcia AO, Otto PI, da Silva MVB, Martins MF, Machado MA et al. Runs of homozygosity and signatures of selection for number of oocytes and embryos in the Gir Indicine cattle. Mamm Genome. 2023:1–15. 10.1007/s00335-023-09989-w 37000236

[18] Xie R, Shi L, Liu J, Deng T, Wang L, Liu Y, Zhao F (2019). Genome-wide scan for runs of homozygosity identifies candidate genes in three pig breeds. Animals.

[19] Pryce JE, Haile-Mariam M, Goddard ME, Hayes BJ (2014). Identification of genomic regions associated with inbreeding depression in Holstein and Jersey dairy cattle. Genet Selection Evol.

[20] Sanglard LP, Huang Y, Gray KA, Linhares DC, Dekkers J, Niederwerder MC (2021). Further host-genomic characterization of total antibody response to PRRSV vaccination and its relationship with reproductive performance in commercial sows: genome-wide haplotype and zygosity analyses. Genet Selection Evol.

[21] Cesarani A, Gaspa G, Pauciullo A, Degano L, Vicario D, Macciotta NP (2021). Genome-wide analysis of homozygosity regions in European simmental bulls. J Anim Breed Genet.

[22] Martikainen K, Koivula M, Uimari P (2020). Identification of runs of homozygosity affecting female fertility and milk production traits in Finnish Ayrshire cattle. Sci Rep.

[23] Ghoreishifar M, Vahedi SM, Salek Ardestani S, Khansefid M, Pryce JE (2023). Genome-wide assessment and mapping of inbreeding depression identifies candidate genes associated with semen traits in Holstein bulls. BMC Genomics.

[24] Nani JP, Peñagaricano F (2020). Whole-genome homozygosity mapping reveals candidate regions affecting bull fertility in US Holstein cattle. BMC Genomics.

[25] Zhao G, Liu Y, Niu Q, Zheng X, Zhang T, Wang Z (2021). Runs of homozygosity analysis reveals consensus homozygous regions affecting production traits in Chinese simmental beef cattle. BMC Genomics.

[26] Davoudi P, Do D, Colombo SM, Rathgeber B, Hu G, Sargolzaei M (2022). Genetic and phenotypic parameters for feed efficiency and component traits in American mink. J Anim Sci.

[27] Do DN, Hu G, Salek Ardestani S, Miar Y (2021). Genetic and phenotypic parameters for body weights, harvest length, and growth curve parameters in American mink. J Anim Sci.

[28] Liu Z, Liu L, Song X, Cong B, Yang F (2017). Heritability and genetic trends for growth and fur quality traits in silver blue mink. Italian J Anim Sci.

[29] Madsen M, Villumsen T, Hansen B, Møller S, Jensen J, Shirali M (2020). Combined analysis of group recorded feed intake and individually recorded body weight and litter size in mink. Animal.

[30] Shirali M, Nielsen V, Møller S, Jensen J (2015). Longitudinal analysis of residual feed intake and BW in mink using random regression with heterogeneous residual variance. Animal.

[31] Purcell S, Neale B, Todd-Brown K, Thomas L, Ferreira MA, Bender D (2007). PLINK: a tool set for whole-genome association and population-based linkage analyses. Am J Hum Genet.

[32] Lencz T, Lambert C, DeRosse P, Burdick KE, Morgan TV, Kane JM et al. Runs of homozygosity reveal highly penetrant recessive loci in schizophrenia. Proceedings of the National Academy of Sciences. 2007;104(50):19942-7. 10.1073/pnas.0710021104 PMC2148402 18077426

[33] Ku CS, Naidoo N, Teo SM, Pawitan Y (2011). Regions of homozygosity and their impact on complex diseases and traits. Hum Genet.

[34] Signer-Hasler H, Henkel J, Bangerter E, Bulut Z, Drögemüller C, Leeb T, Flury C (2022). Runs of homozygosity in Swiss goats reveal genetic changes associated with domestication and modern selection. Genet Selection Evol.

[35] Quinlan AR, Hall IM (2010). BEDTools: a flexible suite of utilities for comparing genomic features. Bioinformatics.

[36] Gezan S, de Oliveira A, Murray D (2021). ASRgenomics: an R package with complementary genomic functions.

[37] VanRaden PM (2008). Efficient methods to compute genomic predictions. J Dairy Sci.

[38] Butler D, Cullis B, Gilmour A, Gogel B, Thompson R. ASReml-R reference manual version 4. VSN International Ltd, Hemel Hempstead, HP1 1ES, UK. 2017.

[39] Karimi K, Ngoc Do D, Sargolzaei M, Miar Y (2021). Population genomics of American mink using whole genome sequencing data. Genes.

[40] Mulim HA, Brito LF, Pinto LFB, Ferraz JBS, Grigoletto L, Silva MR, Pedrosa VB (2022). Characterization of runs of homozygosity, heterozygosity-enriched regions, and population structure in cattle populations selected for different breeding goals. BMC Genomics.

[41] Shi L, Wang L, Liu J, Deng T, Yan H, Zhang L (2020). Estimation of inbreeding and identification of regions under heavy selection based on runs of homozygosity in a large White pig population. J Anim Sci Biotechnol.

[42] Cendron F, Perini F, Mastrangelo S, Tolone M, Criscione A, Bordonaro S (2020). Genome-wide SNP analysis reveals the population structure and the conservation status of 23 Italian chicken breeds. Animals.

[43] Marchesi J, Buzanskas M, Cantão M, Ibelli A, Peixoto J, Joaquim L (2018). Relationship of runs of homozygosity with adaptive and production traits in a paternal broiler line. Animal.

[44] Purfield DC, McParland S, Wall E, Berry DP (2017). The distribution of runs of homozygosity and selection signatures in six commercial meat sheep breeds. PLoS ONE.

[45] Signer-Hasler H, Burren A, Ammann P, Drögemüller C, Flury C (2019). Runs of homozygosity and signatures of selection: a comparison among eight local Swiss sheep breeds. Anim Genet.

[46] Macciotta NP, Colli L, Cesarani A, Ajmone-Marsan P, Low WY, Tearle R, Williams JL (2021). The distribution of runs of homozygosity in the genome of river and swamp buffaloes reveals a history of adaptation, migration and crossbred events. Genet Selection Evol.

[47] Howrigan DP, Simonson MA, Keller MC (2011). Detecting autozygosity through runs of homozygosity: a comparison of three autozygosity detection algorithms. BMC Genomics.

[48] Keller MC, Visscher PM, Goddard ME (2011). Quantification of inbreeding due to distant ancestors and its detection using dense single nucleotide polymorphism data. Genetics.

[49] Gorssen W, Meyermans R, Janssens S, Buys N (2021). A publicly available repository of ROH islands reveals signatures of selection in different livestock and pet species. Genet Selection Evol.

[50] Farid AH, Hussain I, Rupasinghe PP, Stephen J, Arju I (2022). Long-term antibody production and viremia in American mink (Neovison vison) challenged with Aleutian mink disease virus. BMC Vet Res.

[51] Prieto A, Fernández-Antonio R, Díaz-Cao J, López G, Díaz P, Alonso J (2017). Distribution of Aleutian mink disease virus contamination in the environment of infected mink farms. Vet Microbiol.

[52] Farid A, Zillig M, Finley G, Smith G (2012). Prevalence of the Aleutian mink disease virus infection in Nova Scotia. Can Prev Veterinary Med.

[53] Yan Q, Xu R, Zhu L, Cheng X, Wang Z, Manis J, Shipp MA (2013). BAL1 and its partner E3 ligase, BBAP, link poly (ADP-ribose) activation, ubiquitylation, and double-strand DNA repair independent of ATM, MDC1, and RNF8. Mol Cell Biol.

[54] Shen Y, Sun Y, Zhang L, Liu H (2017). Effects of DTX3L on the cell proliferation, adhesion, and drug resistance of multiple myeloma cells. Tumor Biology.

[55] Lo P-K, Yao Y, Lee JS, Zhang Y, Huang W, Kane MA, Zhou Q (2018). LIPG signaling promotes tumor initiation and metastasis of human basal-like triple-negative breast cancer. Elife.

[56] Hong R, Wang Y, Dong H, Geng R (2020). DTX3L/ARTD9 contributes to inflammation of fibroblast-like synoviocytes by increasing STAT1 translocation. Tissue Cell.

[57] Zhu H, Zheng C (2021). When PARPs meet antiviral innate immunity. Trends Microbiol.

[58] Xu H, Chai S, Wang Y, Wang J, Xiao D, Li J, Xiong N (2020). Molecular and clinical characterization of PARP9 in gliomas: a potential immunotherapeutic target. CNS Neurosci Ther.

[59] Xing J, Zhang A, Du Y, Fang M, Minze LJ, Liu Y-J (2021). Identification of poly (ADP-ribose) polymerase 9 (PARP9) as a noncanonical sensor for RNA virus in dendritic cells. Nat Commun.

[60] Mehrotra P, Hollenbeck A, Riley JP, Li F, Patel RJ, Akhtar N, Goenka S (2013). Poly (ADP-ribose) polymerase 14 and its enzyme activity regulates TH2 differentiation and allergic airway disease. J Allergy Clin Immunol.

[61] Cho SH, Raybuck A, Wei M, Erickson J, Nam KT, Cox RG (2013). B cell–intrinsic and–extrinsic regulation of antibody responses by PARP14, an intracellular (ADP-Ribosyl) Transferase. J Immunol.

[62] Liu Y, Liang W-B, Gao L-B, Pan X-M, Chen T-Y, Wang Y-Y (2010). CTLA4 and CD86 gene polymorphisms and susceptibility to chronic obstructive pulmonary disease. Hum Immunol.

[63] Nishimura Y, Shimojima M, Miyazawa T, Sato E, Nakamura K, Izumiya Y (2000). Molecular cloning of the cDNAs encoding the feline B-lymphocyte activation antigen B7-1 (CD80) and B7-2 (CD86) homologues which interact with human CTLA4-Ig. Eur J Immunogenetics: Official J Br Soc Histocompatibility Immunogenet.

[64] Gomez TS, McCarney SD, Carrizosa E, Labno CM, Comiskey EO, Nolz JC (2006). HS1 functions as an essential actin-regulatory adaptor protein at the immune synapse. Immunity.

[65] Adney DR, Lovaglio J, Schulz JE, Yinda CK, Avanzato VA, Haddock E et al. Severe acute respiratory disease in American mink experimentally infected with SARS-CoV-2. JCI Insight. 2022;7(22). 10.1172/jci.insight.159573 PMC9746805 36509288

[66] Enserink M. Coronavirus rips through Dutch mink farms, triggering culls. American Association for the Advancement of Science; 2020. p. 1169. 10.1126/science.368.6496.1169 32527808

[67] Koopmans M (2021). SARS-CoV-2 and the human-animal interface: outbreaks on mink farms. Lancet Infect Dis.

[68] Russo LC, Tomasin R, Matos IA, Manucci AC, Sowa ST, Dale K et al. The SARS-CoV-2 Nsp3 macrodomain reverses PARP9/DTX3L-dependent ADP-ribosylation induced by interferon signaling. J Biol Chem. 2021;297(3). 10.1016/j.jbc.2021.101041 PMC8332738 34358560

[69] Grunewald ME, Chen Y, Kuny C, Maejima T, Lease R, Ferraris D (2019). The coronavirus macrodomain is required to prevent PARP-mediated inhibition of virus replication and enhancement of IFN expression. PLoS Pathog.

[70] Arunachalam PS, Wimmers F, Mok CKP, Perera RA, Scott M, Hagan T (2020). Systems biological assessment of immunity to mild versus severe COVID-19 infection in humans. Science.

[71] Wang F, Hou H, Luo Y, Tang G, Wu S, Huang M et al. The laboratory tests and host immunity of COVID-19 patients with different severity of illness. JCI Insight. 2020;5(10). 10.1172/jci.insight.137799 PMC7259533 32324595

[72] Zhou R, To KK-W, Wong Y-C, Liu L, Zhou B, Li X (2020). Acute SARS-CoV-2 infection impairs dendritic cell and T cell responses. Immunity.

[73] Winheim E, Rinke L, Lutz K, Reischer A, Leutbecher A, Wolfram L (2021). Impaired function and delayed regeneration of dendritic cells in COVID-19. PLoS Pathog.

[74] Wang Y-N, Yang W-C, Li P-W, Wang H-B, Zhang Y-Y, Zan L-S (2018). Myocyte enhancer factor 2A promotes proliferation and its inhibition attenuates myogenic differentiation via myozenin 2 in bovine skeletal muscle myoblast. PLoS ONE.

[75] Juszczuk-Kubiak E, Starzyński RR, Wicińska K, Flisikowski K (2012). Promoter variant-dependent mRNA expression of the MEF2A in longissimus dorsi muscle in cattle. DNA Cell Biol.

[76] Zhou Y, Liu Y, Jiang X, Du H, Li X, Zhu Q (2010). Polymorphism of chicken myocyte-specific enhancer-binding factor 2A gene and its association with chicken carcass traits. Mol Biol Rep.

[77] Chen F, Chen H, Wang J, Niu H, Lan X, Hua L (2010). MEF2A gene polymorphisms are associated with growth traits in Chinese indigenous cattle breeds. J Anim Veterinary Adv.

[78] Foroutan A, Devos J, Wishart DS, Li C, Colazo M, Kastelic J (2021). Impact of prenatal maternal nutrition and parental residual feed intake (RFI) on mRNA abundance of metabolic drivers of growth and development in young Angus bulls. Livest Sci.

[79] Le Goff C, Cormier-Daire V (2011). The ADAMTS (L) family and human genetic disorders. Hum Mol Genet.

[80] Metzger J, Rau J, Naccache F, Bas Conn L, Lindgren G, Distl O (2018). Genome data uncover four synergistic key regulators for extremely small body size in horses. BMC Genomics.

[81] Lee Y-L, Bosse M, Mullaart E, Groenen MA, Veerkamp RF, Bouwman AC (2020). Functional and population genetic features of copy number variations in two dairy cattle populations. BMC Genomics.

[82] Hoopes BC, Rimbault M, Liebers D, Ostrander EA, Sutter NB (2012). The insulin-like growth factor 1 receptor (IGF1R) contributes to reduced size in dogs. Mamm Genome.

[83] Bannasch DL, Baes CF, Leeb T (2020). Genetic variants affecting skeletal morphology in domestic dogs. Trends Genet.

[84] Van Duyvenvoorde HA, Lui JC, Kant SG, Oostdijk W, Gijsbers AC, Hoffer MJ (2014). Copy number variants in patients with short stature. Eur J Hum Genet.

[85] Gudbjartsson DF, Walters GB, Thorleifsson G, Stefansson H, Halldorsson BV, Zusmanovich P (2008). Many sequence variants affecting diversity of adult human height. Nat Genet.

[86] Lettre G, Jackson AU, Gieger C, Schumacher FR, Berndt SI, Sanna S (2008). Identification of ten loci associated with height highlights new biological pathways in human growth. Nat Genet.

[87] Frischknecht M, Flury C, Leeb T, Rieder S, Neuditschko M (2016). Selection signatures in Shetland ponies. Anim Genet.

[88] Peripolli E, Reimer C, Ha N-T, Geibel J, Machado MA, Panetto JCC (2020). Genome-wide detection of signatures of selection in indicine and Brazilian locally adapted taurine cattle breeds using whole-genome re-sequencing data. BMC Genomics.

[89] Lin Y-MJ, Hsin I-L, Sun HS, Lin S, Lai Y-L, Chen H-Y (2018). NTF3 is a novel target gene of the transcription factor POU3F2 and is required for neuronal differentiation. Mol Neurobiol.

[90] Westphal DS, Riedhammer KM, Kovacs-Nagy R, Meitinger T, Hoefele J, Wagner M (2018). A de novo missense variant in POU3F2 identified in a child with global developmental delay. Neuropediatrics.

[91] Schönauer R, Jin W, Findeisen C, Valenzuela I, Devlin LA, Murrell J (2023). Monoallelic intragenic POU3F2 variants lead to neurodevelopmental delay and hyperphagic obesity, confirming the gene’s candidacy in 6q16. 1 deletions. Am J Hum Genet.

[92] Rizkalla SW, Prifti E, Cotillard A, Pelloux V, Rouault C, Allouche R (2012). Differential effects of macronutrient content in 2 energy-restricted diets on cardiovascular risk factors and adipose tissue cell size in moderately obese individuals: a randomized controlled trial. Am J Clin Nutr.

[93] Sun Y, Zhao G, Liu R, Zheng M, Hu Y, Wu D (2013). The identification of 14 new genes for meat quality traits in chicken using a genome-wide association study. BMC Genomics.

[94] Vigors S, O’Doherty JV, Bryan K, Sweeney T (2019). A comparative analysis of the transcriptome profiles of liver and muscle tissue in pigs divergent for feed efficiency. BMC Genomics.

[95] NFACC. Code of practice for the care and handling of farmed mink. National Farm Animal Care Council Lacombe, Alberta; 2013.

